# Effects of streptozotocin-induced diabetes on the pharmacology of rat conduit and resistance intrapulmonary arteries

**DOI:** 10.1186/1475-2840-8-4

**Published:** 2009-01-21

**Authors:** Alison M Gurney, Frank C Howarth

**Affiliations:** 1 Faculty of Life Sciences, University of Manchester, Floor 2, Core Technology Facility, 46 Grafton Street, Manchester, M13 9NT, UK; 2 Department of Physiology, Faculty of Medicine & Health Sciences, United Arab Emirates University, P.O. Box 17666, Al Ain, UAE

## Abstract

**Background:**

Poor control of blood glucose in diabetes is known to promote vascular dysfunction and hypertension. Diabetes was recently shown to be linked to an increased prevalence of pulmonary hypertension. The aim of this study was to determine how the pharmacological reactivity of intrapulmonary arteries is altered in a rat model of diabetes.

**Methods:**

Diabetes was induced in rats by the β-cell toxin, streptozotocin (STZ, 60 mg/kg), and isolated conduit and resistance intrapulmonary arteries studied 3–4 months later. Isometric tension responses to the vasoconstrictors phenylephrine, serotonin and PGF_2α_, and the vasodilators carbachol and glyceryl trinitrate, were compared in STZ-treated rats and age-matched controls.

**Results:**

STZ-induced diabetes significantly blunted the maximum response of conduit, but not resistance pulmonary arteries to phenylephrine and serotonin, without a change in pEC_50_. Agonist responses were differentially reduced, with serotonin (46% smaller) affected more than phenylephrine (32% smaller) and responses to PGF_2α _unaltered. Vasoconstriction caused by K^+^-induced depolarisation remained normal in diabetic rats. Endothelium-dependent dilation to carbachol and endothelium-independent dilation to glyceryl trinitrate were also unaffected.

**Conclusion:**

The small resistance pulmonary arteries are relatively resistant to STZ-induced diabetes. The impaired constrictor responsiveness of conduit vessels was agonist dependent, suggesting possible loss of receptor expression or function. The observed effects cannot account for pulmonary hypertension in diabetes, rather the impaired reactivity to vasoconstrictors would counteract the development of pulmonary hypertensive disease.

## Background

Hypertension is a recognised consequence of the poor control of blood glucose in diabetes. A recent study found that patients with diabetes mellitus also have an increased prevalence of pulmonary hypertension, independent of systemic hypertension, coronary artery disease, congestive heart failure or smoking [1]. In addition, pulmonary hypertension is more severe in patients with chronic obstructive pulmonary disease if they also have diabetes [2]. Furthermore, maternal diabetes is a risk factor for persistent pulmonary hypertension of the newborn [3]. Thus uncontrolled diabetes is a contributor to the development of pulmonary vascular disease.

The underlying cause of systemic hypertension in diabetes is thought to be peripheral vasoconstriction [4] mediated, at least in part, by endothelial dysfunction [5,6] and enhanced smooth muscle contractility [7]. Little is known about the cause of pulmonary hypertension, but there is evidence from a rat model of diabetes, induced by streptozotocin (STZ) [8], that uncontrolled plasma glucose gives rise to structural [9] and biochemical [10] changes in pulmonary arteries. A study of salt-perfused rat lungs found that pulmonary vascular resistance was elevated in male, but not female rats subject to STZ-induced diabetes, along with reduced reactivity of the pulmonary vessels to vasoconstrictors [11]. A recent report suggests that, as in systemic vessels, STZ-induced diabetes in rats leads to loss of endothelium-dependent relaxation in the main intrapulmonary artery, possibly reflecting enhanced superoxide production from NADPH oxidase [12].

A single injection of STZ is widely used to generate a rat model of type I diabetes, which results from the selective toxicity of STZ towards the insulin-producing β-cells in pancreatic islets [13]. A number of factors influence the vascular dysfunction that develops in this model, such as the age of the rats, the dose of STZ administered and the duration and severity of hyperglycaemia [8]. For example, the endothelium-dependent relaxation of rat aorta was enhanced 24 hrs after STZ injection, normal at 1–2 weeks and depressed at 8 weeks [6]. Impaired endothelial function was also more common in young adult humans with a longer duration of diabetes [14]. There may also be differential effects of diabetes on different blood vessels. For example, mesenteric arteries from STZ-treated rats displayed an enhanced vasoconstrictor response to noradrenaline and reduced sensitivity to endothelium-dependent vasodilators at a time (2–10 weeks after STZ injection) when aortae from the same animals responded normally [15].

Since the small resistance arteries in the lung are the main determinants of pulmonary vascular resistance and pulmonary arterial pressure, the aim of this study was to determine how chronic diabetes (3–4 months) affects these vessels. Reactivity to vasoconstrictors and vasodilators was investigated in intrapulmonary arteries (IPA) of ≤250 μm in diameter and compared with larger conduit arteries from the same lungs, in STZ diabetic animals and age-matched controls.

## Methods

Diabetes was induced in young male Wistar rats (200–250 g) by a single intraperitoneal injection of 60 mg/kg STZ (Sigma) dissolved in citrate buffer, containing 100 mM citric acid and 100 mM sodium citrate at pH 4.5. This dose of STZ lies within the range used in most cardiovascular studies and produces moderate diabetes, in which blood glucose levels are 3–4 times normal, by causing substantial but incomplete depletion of pancreatic insulin [16]. Age-matched controls received an equivalent volume of vehicle. Control and STZ-treated animals were maintained on the same diet with freely available water. Animals were sacrificed at 3–4 months immediately after measuring body weight and blood glucose. A blood glucose level exceeding 200 mg/dL was considered diabetic. The lungs were quickly removed into physiological salt solution (PSS) of the following composition (mM): NaCl 112, KCl 5, KH_2_PO_4 _0.5, Na_2_HPO_4 _0.5, CaCl_2 _1.8, MgCl_2 _1, HEPES 10, glucose 11; pH7.3.

The investigation was approved by the ethics committee of the Faculty of Medicine & Health Sciences, United Arab Emirates University and conforms to the Guide *for the Care and Use of Laboratory Animals *published by the National Institutes of Health (NIH Publication No. 85-23, revised 1996).

### Small vessel myography

A section of conduit artery (~500 μm diameter), from half way down the main artery running the length of the left lung lobe, was dissected out and mounted between two pins in a small vessel myograph chamber (Danish Myotechnology). Resistance-sized intrapulmonary arteries (< 250 μm diameter) were dissected from the same lobe and mounted between two 40 μm wires in separate myograph chambers. The vessels were bathed in PSS continually aerated at 37°C. A basal tension of 5 mN (conduit vessels) or 4 mN (resistance vessels) was applied and the vessels allowed to equilibrate for 30 min, washing at 15 min intervals. At the start of each experiment, 50 mM KCl was applied to each vessel until a maximal contractile response was observed. The vessels were then washed until tension returned to baseline and the challenge with high K^+ ^repeated until reproducible contractions were recorded.

The sensitivity of intrapulmonary arteries to depolarisation-induced constriction was determined by replacing the PSS in the myograph chamber with PSS in which the K^+ ^concentration was sequentially increased to 15 mM, 30 mM, 50 mM, 70 mM, 90 mM or 110 mM, by equimolar substitution of KCl for NaCl. To determine sensitivity to vasoconstrictors, vessels were exposed to increasing concentrations of phenylephrine (0.1 nM – 100 μM), serotonin (1 nM – 100 μM) or prostaglandin F_2α _(PGF_2α_; 1 nM – 10 μM), applied cumulatively with at least 5 min between additions. The contractile force generated by the different constrictor agents was measured as a percentage of the force generated by 50 mM KCl, which evoked a maximum contractile response of 2.1 ± 0.2 mN (n = 21) and 2.8 ± 0.3 mN (n = 25) in large arteries from control and STZ-treated animals, respectively, compared with 4.0 ± 0.5 mN (n = 23) and 5.1 ± 0.9 mN (n = 23) in the small arteries from control and STZ-treated animals. The maximum response and the concentration of drug causing 50% of the maximum response (EC_50_) were determined by fitting the Hill equation to the dose-response curves for each vessel.

To determine the sensitivity of intrapulmonary arteries to vasodilator drugs, vessels were pre-constricted to near the maximum response with a vasoconstrictor. Once a plateau level of tension was reached, increasing concentrations of carbachol (1 nM – 100 μM) or glyceryl trinitrate (GTN; 1 nM – 100 μM) were applied cumulatively, with at least 5 min between additions. Phenylephrine (100 nM or 1 μM) was used as the vasoconstrictor for conduit vessels. Resistance vessels showed small and variable responses to phenylephrine, and agonist-induced constriction was not always sustained sufficiently to allow dilator effects to be assessed. Individual resistance vessels were therefore pre-constricted with whichever agent was found to cause the most substantial and sustained constriction.

### Data Analysis

Results are presented as mean ± S.E.M, with 'n' referring to the number of rats used. Statistical analysis was performed using Origin 7.5 (Origin Lab) or Excel 2003 (Microsoft) software. A paired t-test was used to compare parameters in conduit and resistance arteries from the same animals. Otherwise an independent t-test was used for statistical comparisons. P < 0.05 was taken to indicate significance.

### Drugs

Glyceryl trinitrate (nitrocine) was obtained from Schwarz Pharma as a 1 mg/ml solution. PGF_2α _(tromethamine salt), from Cayman Chemicals, was prepared as a 100 mM stock solution in dimethylsulphoxide. Phenylephrine hydrochloride, serotonin creatinine sulphate and carbamylcholine chloride (carbachol) were from Sigma and prepared as 10 mM stock solutions in distilled water. Aliquots of each stock solution were stored frozen and diluted in PSS as required.

## Results

The control rats had a mean body weight of 355 ± 9 g and blood glucose of 82 ± 6 mg/dl (n = 23). The diabetic rats had significantly lower body weight (266 ± 5 g, p < 0.0001) and higher blood glucose levels (319 ± 35 mg/dl, p < 0.0001) than the age-matched control animals.

### Depolarisation induced vasoconstriction

**Figure 1:**
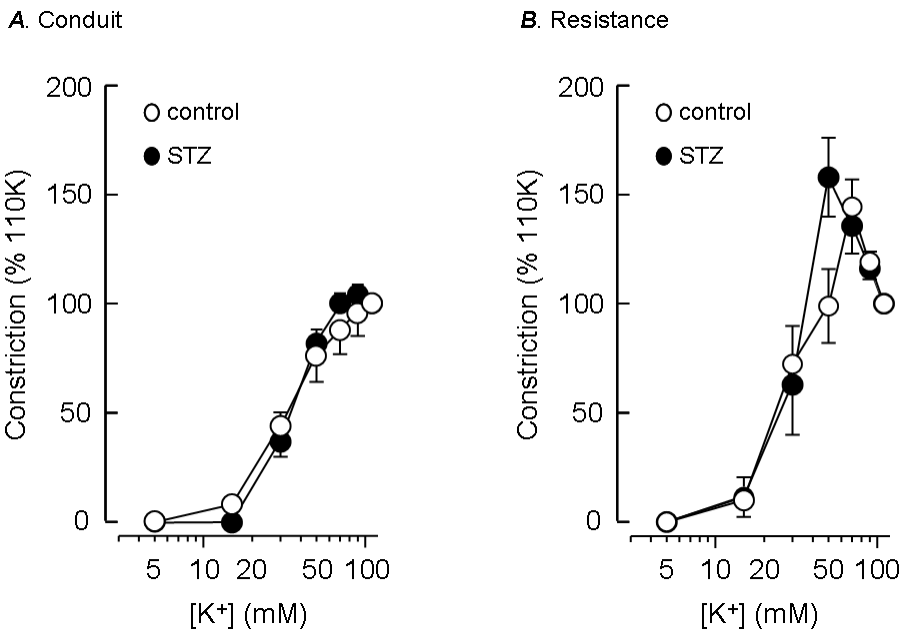
**Resistance of depolarisation-induced pulmonary artery constriction to STZ-induced diabetes**. Constrictor responses to increasing concentrations of extracellular K^+^, measured as a percentage of the response to 110 mM K^+^, in conduit (A) and resistance-sized (B) intrapulmonary arteries from STZ-treated (solid circles) and age-matched control (open circles) rats. Data plotted are mean ± s.e.m. of 8–10 animals.

Elevation of the extracellular K^+ ^concentration causes constriction of pulmonary arteries due to depolarisation of the smooth muscle membrane [17] and voltage-gated Ca^2+ ^influx. Figure 1 compares the concentration dependence of K^+^-induced constriction of conduit (A) and resistance (B) intrapulmonary arteries from STZ-treated rats and age-matched controls. STZ-induced diabetes had little effect on the concentration-response curve to K^+ ^in small or large vessels. The maximum response of conduit arteries was observed above 50 mM K^+^, with 50% of the maximum seen at 42 ± 8 mM (n = 8) in control vessels and 36 ± 4 mM (n = 10) in vessels from STZ-treated rats, with no significant difference between these values. The K^+ ^concentration-response relationship in resistance arteries reached a maximum between 50 and 70 mM K^+ ^and declined at higher concentrations. A similar maximum constriction was seen in the small vessels from control and STZ-treated animals, amounting to 164 ± 18% (n = 9) and 151 ± 12% (n = 8) of the response to 110 mM K^+^, respectively. The K^+ ^concentration producing a half maximal response was also similar in resistance vessels from STZ-treated (30 ± 4 mM) and control (36 ± 5 mM) arteries. These values were not significantly different from the K^+ ^concentrations producing 50% of the maximal response in conduit vessels.

### Responses to vasoconstrictor receptor agonists

**Figure 2:**
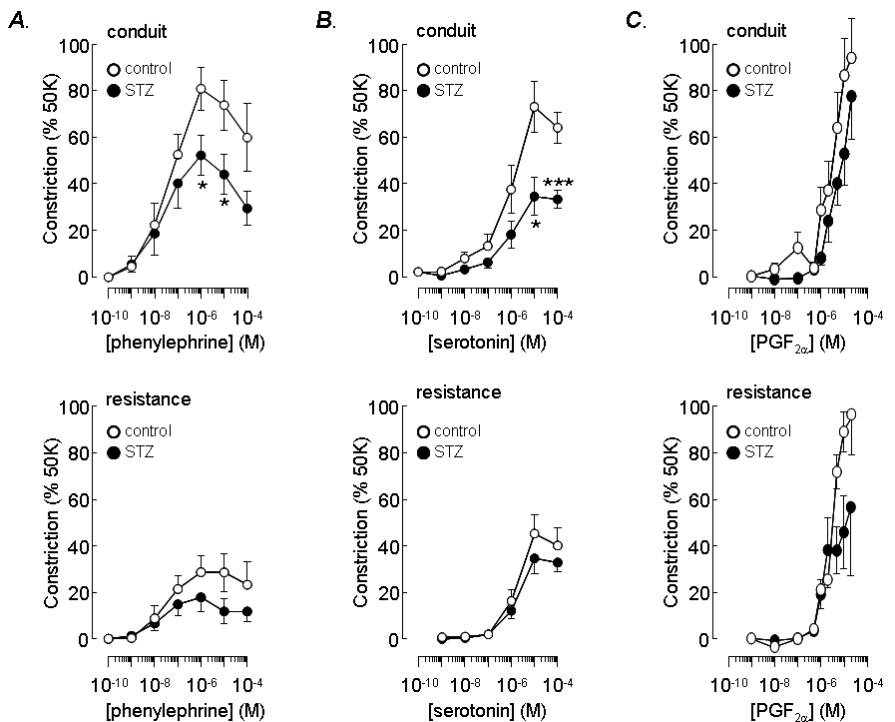
**Effect of STZ-induced diabetes on responses to vasoconstrictor agonists**. Concentration response curves for vasoconstriction evoked by phenylephrine (A), serotonin (B) and PGF_2α _(C) in conduit (upper panels) and resistance (lower panels) arteries. Responses were measured as a percentage of the constriction induced by 50 mM KCl and are shown for STZ-treated, diabetic (solid circles) and age-matched control rats. Data plotted are mean ± s.e.m. with n = 12–13 (phenylephrine), n = 12 (serotonin) or n = 3–10 (PGF_2α_). *P < 0.05, ***P < 0.001 versus control.

The effects of three receptor agonists, widely used in physiological studies of pulmonary artery, were investigated in intrapulmonary arteries from control and STZ-treated rats. Phenylephrine, an α_1_-adrenoceptor agonist, serotonin and prostaglandin F_2α _(PGF_2α_) all produced concentration-dependent constriction of conduit and resistance vessels from control and diabetic animals (Figure 2). The concentration-response curves for phenylephrine displayed a peak at 1 μM, waning at higher concentrations (Figure 2A). We did not investigate the falling phase further and responses to the highest concentrations of agonist were ignored when fitting the Hill equation to estimate the maximum response and pEC_50 _values for each tissue.

As previously reported [18], resistance vessels were significantly less responsive to phenylephrine than conduit vessels. While all of the conduit vessels tested from control animals showed a robust response to phenylephrine, several resistance vessels displayed either little or no response. The mean maximum constriction measured in control conduit vessels, at 84 ± 9% (n = 12) of the response to 50 mM KCl, was significantly larger than that measured in control resistance arteries (33 ± 8%, n = 13, p < 0.001). There was a similar difference between the maximum responses to phenylephrine measured in conduit (57 ± 8%, n = 12) and resistance (21 ± 6%, n = 13, p < 0.01) vessels from STZ-treated animals. From Figure 2A it can be seen that STZ treatment resulted in a loss of phenylephrine efficacy, with the maximum response of conduit vessels 32% smaller (p < 0.05) in the STZ-treated animals. Resistance vessels from STZ-treated rats appeared to show a 36% smaller maximum response relative to the age-matched controls, but this difference did not reach statistical significance. In contrast to the effect of STZ-induced diabetes on the maximum response to phenylephrine, it had no effect on the pEC_50_, which was the same in all vessels: 7.4 ± 0.2 (n = 12) in control conduit arteries, 7.5 ± 0.2 (n = 11) in STZ-treated conduit arteries, 7.5 ± 0.2 (n = 13) in control resistance vessels and 7.2 ± 0.2 (n = 12) in STZ-treated resistance arteries.

Resistance arteries from control animals were also less sensitive to serotonin than conduit arteries from the same animals (Figure 2B), with maximum responses amounting to 85 ± 10% (n = 12) of the KCl constriction in conduit vessels and 56 ± 9% (n = 12, p < 0.05) in resistance arteries. This differential effect disappeared in vessels from STZ-treated rats, where serotonin produced a maximum response of 46 ± 7% (n = 12) in conduit arteries and 51 ± 7% (n = 12) in resistance vessels. These results indicate that in STZ-induced diabetes, conduit vessels experienced a 46% (p < 0.01) reduction in the maximum response to serotonin while the response of resistance vessels was essentially unchanged, with the measured 9% reduction failing to reach statistical significance. The pEC_50 _values for serotonin-induced constriction were similar in conduit arteries from control (5.8 ± 0.2, n = 12) and STZ-treated (5.6 ± 0.2, n = 12) animals and in resistance arteries from control (5.55 ± 0.08, n = 12) and STZ-treated (5.4 ± 0.2, n = 12) animals.

In contrast to the results with phenylephrine and serotonin, conduit and resistance arteries showed a similar sensitivity to PGF_2α_-induced vasoconstriction (Figure 2C). A maximum response was not always reached at the highest concentration of PGF_2α _tested. Nevertheless, at 10 μM PGF_2α_, which appeared to produce a near maximum contraction, there was no significant difference between the responses evoked in conduit (86 ± 16%, n = 6) or resistance (89 ± 18%, n = 7) arteries from control animals. The responses of conduit (53 ± 13%, n = 5) and resistance arteries (46 ± 15%, n = 5) from STZ-treated animals to 10 μM PGF_2α _were also not significantly different. Furthermore, STZ had little effect on the sensitivity of intrapulmonary arteries to PGF_2α_. Although responses to 10 μM PGF_2α _appeared smaller in conduit (38%) and resistance (48%) arteries from animals treated with STZ compared with the age-matched controls, these differences were not statistically significant. Estimates of pEC_50 _were also not significantly different between control (conduit 5.5 ± 0.2, n = 8; resistance 5.57 ± 0.05, n = 7) and STZ-treated (conduit 5.59 ± 0.07, n = 5; resistance 5.66 ± 0.08, n = 7) animals.

### Responses to vasodilator drugs

**Figure 3:**
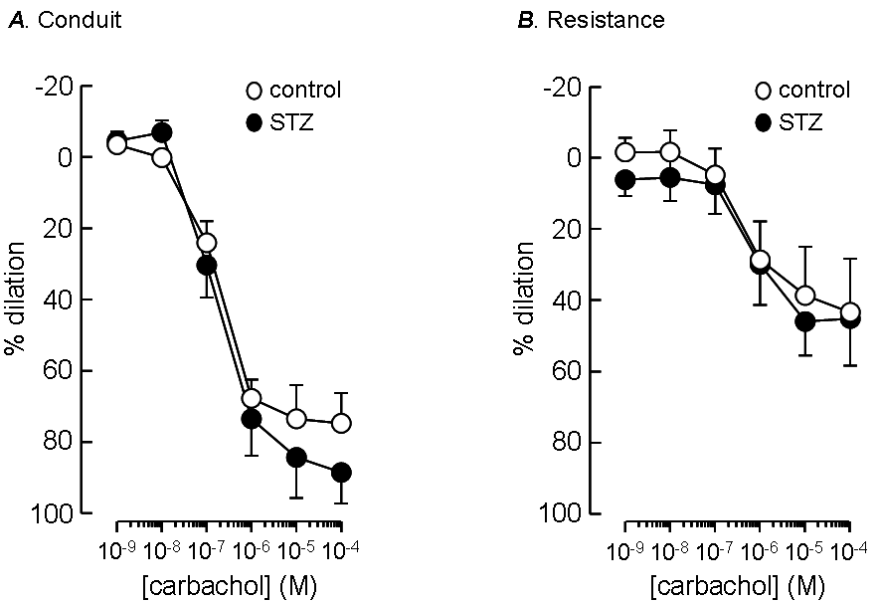
**Resistance of endothelium-dependent vasodilation to STZ-induced diabetes**. Dilator responses to increasing concentrations of carbachol, measured as a percentage of pre-constrictor tone, in conduit (A) and resistance-sized (B) intrapulmonary arteries from STZ-treated (solid circles) and age-matched control (open circles) rats. Data plotted are mean ± s.e.m. of 9–11 animals.

Conduit and resistance arteries from control and STZ-treated animals displayed a robust response to the endothelium-dependent vasodilator, carbachol (Figure 3), indicating that the integrity of the endothelium was well preserved during vessel preparation even in the small arteries. The dilator effect of carbachol, measured relative to the constrictor tone present before its application, was however smaller in resistance arteries compared with conduit vessels. The vessels from control animals responded with a maximum dilation of 75 ± 8% (n = 9) in conduit arteries and 44 ± 15% (n = 9, p = 0.05) in resistance arteries. Vessels from STZ-treated, diabetic animals showed a similar difference, conduit arteries responding with a maximum relaxation of 88 ± 9% (n = 9) compared with 46 ± 10% (n = 9, p < 0.05) in resistance arteries. It is apparent from Figure 3 that there was no effect of STZ on the responses to carbachol, the maximum responses being similar in the equivalent vessels from STZ-treated and age-matched control animals. Similar pEC_50 _values for carbachol induced vasodilation were found in conduit arteries from control (7.0 ± 0.2, n = 9) and STZ-treated (6.7 ± 0.2, n = 9) animals and in resistance arteries from control (6.8 ± 0.4, n = 8) and STZ-treated (6.9 ± 0.4, n = 7) animals.

**Figure 4:**
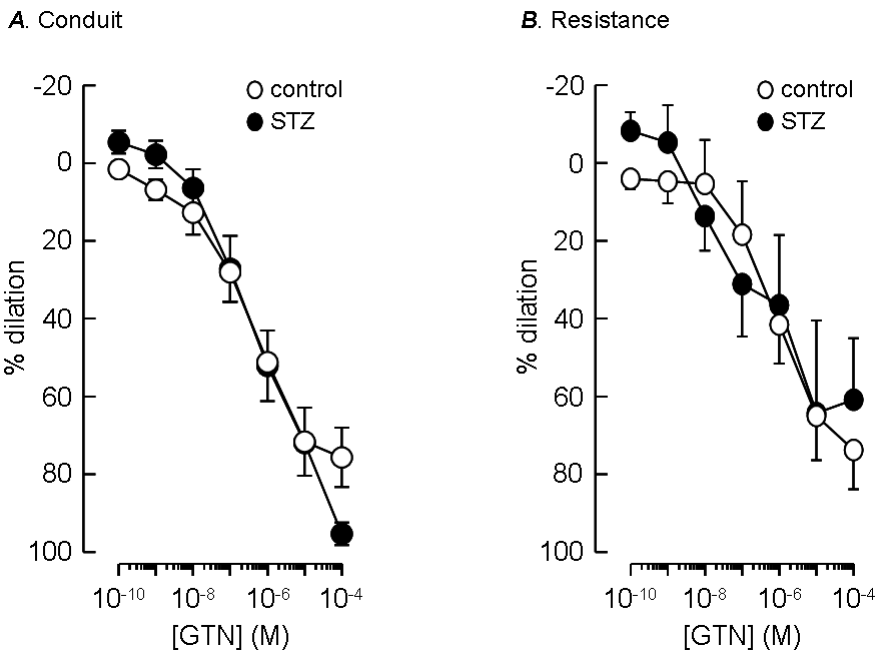
**Resistance of endothelium-independent vasodilation to STZ-induced diabetes**. Dilator responses to increasing concentrations of glyceryl trinitrate (GTN), measured as a percentage of pre-constrictor tone, in conduit (A) and resistance-sized (B) intrapulmonary arteries from STZ-treated (solid circles) and age-matched control (open circles) rats. Data plotted are mean ± s.e.m. of 8–9 animals.

Glyceryl trinitrate (GTN) was used as an example of a directly acting vasodilator, which releases nitric oxide in pulmonary artery smooth muscle cells to activate cGMP-dependent relaxation [19]. Conduit and resistance pulmonary arteries appeared to be equally sensitive to GTN, which evoked dilation with similar potency in all arteries, whether isolated from control or STZ-treated animals (Figure 4). In vessels from control animals, the maximum vasodilator response to GTN, measured relative to the constrictor tone present before its application, was 72 ± 6% (n = 8) in conduit arteries and 80 ± 8% (n = 9) in resistance arteries. The maximum dilation response induced by GTN in vessels from STZ-treated animals was 83 ± 3% (n = 8) in conduit arteries and 86 ± 21% (n = 7) in resistance arteries. The pEC_50 _values for the GTN-induced dilation of conduit vessels were 6.7 ± 0.3 (n = 8) for control animals and 6.6 ± 0.3 (n = 8) for STZ-treated diabetic animals. Similar pEC_50 _values were obtained in resistance arteries, with 6.6 ± 0.5 (n = 9) found for control animals and 7.3 ± 0.5 (n = 7) for STZ-treated diabetic animals. In the studies on GTN action there were no significant differences found between any of the measurements of pEC_50 _or maximum response.

## Discussion

The results imply that rat small intrapulmonary arteries are relatively resistant to diabetes, even 3–4 months after induction by STZ. Depolarisation-induced vasoconstriction was well maintained in both conduit and resistance intrapulmonary arteries. Agonist induced vasoconstriction was not significantly altered by diabetes in resistance arteries, but it was blunted in conduit vessels. There are several reasons to believe that this effect was due to hyperglycaemia rather than a direct action of STZ. Firstly, loss of the pulmonary pressor response to agonist in the salt-perfused lung of STZ diabetic rats was prevented by intensive insulin therapy [11,20]. In addition, PAs from rats treated with a higher dose of STZ and studied 6 weeks after exposure did not display a loss of responsiveness to phenylephrine [12]. Surprisingly, endothelium-dependent vasodilation as well as endothelium-independent vasodilation appeared normal in the 3–4 month diabetic animals. Thus there was no evidence for endothelial dysfunction in the pulmonary arteries of diabetic rats in this study.

Another study recently examined the effects of STZ-induced diabetes on isolated rat pulmonary arteries, with differing results. Lopez-Lopez and colleagues found that STZ treatment enhanced the constrictor response to phenylephrine, while reducing the endothelium-dependent relaxation to acetylcholine [12]. There are several possible reasons for the discrepancies. Firstly the latter study employed larger arteries (0.5–0.8 mm) than used here and our results suggest that larger pulmonary arteries are more sensitive to the effects of diabetes. It also used smaller rats and a larger dose of STZ, which may have resulted in more severe hyperglycaemia [8]. This is further suggested by the criterion for hyperglycaemia (>350 mg/dL), which was higher than applied here, although mean blood glucose levels were not reported. On the other hand, arteries were examined after only 6 weeks of diabetes compared with 3–4 months here. Since the effects of diabetes on the systemic vasculature are known to change over time [6,21] it is possible that an early loss of endothelium-dependent vasodilation in the pulmonary circulation is compensated for later in the disease.

### Vasoconstriction

Conduit and resistance pulmonary arteries showed distinct K^+ ^concentration-response relationships, the maximum contractile force being maintained at high K^+ ^concentrations in conduit but not resistance arteries. The drop off in the response of small vessels at high K^+ ^concentrations is likely to reflect cellular events downstream of membrane depolarisation, because the smooth muscle membrane potentials in small and large pulmonary arteries show a similar dependence on K^+ ^concentration [22]. Whatever the reason for the different sensitivities of conduit and resistance vessels to K^+^, the relationships were unaffected by STZ-induced diabetes, implying that depolarisation-induced Ca^2+ ^entry and the pathways linking the rise in smooth muscle Ca^2+ ^concentration to contraction were well maintained in this model of diabetes.

Conduit arteries displayed a 61% larger maximum response to phenylephrine and a 34% larger maximum response to serotonin compared with resistance vessels, consistent with previous studies on similar sized pulmonary arteries [18,23]. The differential effect of serotonin disappeared in the STZ diabetic rats, apparently due to a greater loss of responsiveness in the conduit than resistance arteries. The conduit arteries from diabetic rats also displayed a significant loss of responsiveness to phenylephrine. The maximum response of resistance arteries to phenylephrine was 64% smaller than in the conduit vessels, but was not significantly altered in diabetes. Thus it appears that diabetes preferentially blunts responsiveness to agonist-induced constriction in the conduit vessels. A similar impairment of phenylephrine and serotonin induced constriction was seen in aortae from rats experiencing a similar duration of STZ-induced diabetes [24], although other studies found the phenylephrine sensitivity of aorta to be unchanged or enhanced [e.g. [15,25]].

In contrast to our findings with phenylephrine and serotonin, but consistent with a previous report [18], PGF_2α _produced similar constrictor responses in conduit and resistance pulmonary arteries. Moreover, the responses in both vessels were not significantly affected by STZ diabetes. The effect of chronic diabetes on vasoconstrictor responses was therefore agonist dependent, the order of sensitivity being serotonin > phenylephrine > PGF_2α_, and it was largely restricted to the conduit pulmonary arteries. Although the agonist responsiveness of resistance arteries was not significantly altered in diabetic rats, the maximum constriction to all three agents tested did show a trend towards a decrease. Perhaps this reflects the early stages of changes in vascular reactivity that might become significant in more severe or prolonged diabetes. The agonist dependence of the deficit in conduit arteries suggests that it reflects loss of receptors or reduced receptor responsiveness, rather than altered downstream pathways. Since diabetes did not affect the pEC_50 _values of any agonist, it seems likely that there is altered receptor activity without a change in the molecular makeup of the receptors. Further experiments, such as measurements of receptor binding and evoked changes in cytoplasmic Ca^2+ ^concentration, will be required to determine the underlying mechanism.

The comparatively greater effect of diabetes on serotonin-induced vasoconstriction is interesting in view of evidence linking enhanced serotonergic activity, both at serotonin receptors and the serotonin transporter, to pulmonary hypertension [26,27]. Serum serotonin concentrations are elevated in diabetics [28] and there is evidence for up regulation of the serotonin transporter in platelets [29] and brain [30]. These effects may well contribute to the increased prevalence of pulmonary hypertension in diabetics, because over expression of the serotonin transporter in mice causes pulmonary hypertension [31]. The loss of responsiveness of pulmonary arteries to serotonin in diabetes would have the opposite effect. So perhaps this change reflects a compensatory mechanism, which arises to counteract the increased activity of peripheral serotonin pathways and protect against the development of pulmonary vasoconstriction.

### Vasodilation

The STZ diabetic rats in this study displayed normal endothelium-dependent dilation to carbachol in both conduit and resistance pulmonary arteries. This contrasts with many studies reporting impaired endothelium-dependent dilation in systemic arteries. There are, however, mixed reports from studies examining endothelium-dependent dilation of aorta after 3 months of STZ-induced diabetes. While impaired responses have been reported [24], others found no effect on the endothelium [25,32], a longer period of diabetes (52 weeks) being required to produce a deficit [25]. On the other hand, loss of endothelium-dependent vasodilation has been reported in rat aorta following shorter periods of diabetes [6,33,34] and as early as 2 weeks after STZ treatment [35,36]. Similar disparities can be found among studies on renal [37], mesenteric [38] and coronary [39] arteries. The reasons for these discrepancies are not clear, but could be related to the dose of STZ administered and/or the age and gender of the rats used, as all of these factors affect the severity and time course of hyperglycaemia [8].

Although there are inconsistencies in the literature concerning the effects of STZ-induced diabetes on responses of systemic arteries to vasoconstrictors and endothelium-dependent vasodilators, there seems to be widespread agreement that STZ treatment does not affect responses to vasodilators that act directly on smooth muscle, such as GTN and sodium nitroprusside. The effect of GTN on pulmonary arteries was also unaffected by chronic diabetes. Thus enhanced dilator potency of nitric oxide, released constitutively from the endothelium, cannot explain the loss of responsiveness to phenylephrine and serotonin in pulmonary arteries from diabetic rats.

## Conclusion

The results of this study suggest that small resistance pulmonary arteries are relatively resistant to STZ-induced diabetes. While conduit arteries showed a loss of responsiveness to serotonin and phenyephrine in chronic diabetes, no significant change was seen in resistance arteries. Diabetes had a differential effect on the constrictor responses to different receptor agonists, with serotonin affected more than phenylephrine and PGF_2α _constriction unchanged, suggesting that there may be differentially altered expression or function of receptors. The loss of agonist responses may represent a compensatory mechanism to protect the pulmonary circulation from changes in diabetes that promote pulmonary vasoconstriction. In this study, chronic diabetes had no effect on endothelium-dependent or endothelium-independent vasodilation, but an effect on the endothelium cannot be ruled out as a recent study, carried out under different conditions, did find reduced endothelium-dependent vasodilatation in conduit pulmonary arteries.

## Abbreviations

STZ: streptozotocin; IPA: intrapulmonary artery; PSS: physiological salt solution; PGF_2α_: prostaglandin F_2α_; GTN: glyceryl trinitrate; HEPES: 4-(2-hydroxyethyl)piperazine-1-ethanesulfonic acid.

## Competing interests

The authors declare that they have no competing interests.

## Authors' contributions

AG carried out the myography experiments, performed the analysis and drafted the manuscript. FH provided the STZ rat model of diabetes and contributed to revision of the manuscript. Both authors participated in the conception and design of the study and approved the final manuscript.

## References

[B1] Movahed MR, Hashemzadeh M, Jamal MM (2005). The prevalence of pulmonary embolism and pulmonary hypertension in patients with type II diabetes mellitus. Chest.

[B2] Makarevich AE, Valevich VE, Pochtavtsev AU (2007). Evaluation of pulmonary hypertension in COPD patients with diabetes. Adv Med Sci.

[B3] Van Marter LJ, Leviton A, Allred EN, Pagano M, Sullivan KF, Cohen A, Epstein MF (1996). Persistent pulmonary hypertension of the newborn and smoking and aspirin and nonsteroidal antiinflammatory drug consumption during pregnancy. Pediatrics.

[B4] Brands MW, Fitzgerald SM (2001). Arterial pressure control at the onset of type I diabetes: the role of nitric oxide and the renin-angiotensin system. Am J Hypertens.

[B5] Johnstone MT, Creager SJ, Scales KM, Cusco JA, Lee BK, Creager MA (1993). Impaired endothelium-dependent vasodilation in patients with insulin-dependent diabetes mellitus. Circulation.

[B6] Pieper GM (1999). Enhanced, unaltered and impaired nitric oxide-mediated endothelium-dependent relaxation in experimental diabetes mellitus: importance of disease duration. Diabetologia.

[B7] Okon EB, Szado T, Laher I, McManus B, van Breemen C (2003). Augmented contractile response of vascular smooth muscle in a diabetic mouse model. J Vasc Res.

[B8] Bell RH, Hye RJ (1983). Animal models of diabetes mellitus: physiology and pathology. J Surg Res.

[B9] Reinilä A (1981). Long-term effects of untreated diabetes on the arterial wall in rat. An ultrastructural study. Diabetologia.

[B10] Reinilä A, Koivisto VA, Akerblom HK (1977). Lipids in the pulmonary artery and the lungs of severely diabetic rats. A histochemical and chemical study. Diabetologia.

[B11] Russ RD, Tobin BW (1998). Differential pulmonary vascular effects of streptozotocin diabetes in male and female rats. Proc Soc Exp Biol Med.

[B12] Lopez-Lopez JG, Moral-Sanz J, Frazziano G, Gomez-Villalobos MJ, Flores-Hernandez J, Monjaraz E, Cogolludo A, Perez-Vizcaino F (2008). Diabetes induces pulmonary artery endothelial dysfunction by NADPH oxidase induction. Am J Physiol.

[B13] Rerup CC (1970). Drugs producing diabetes through damage of the insulin secreting cells. Pharmacol Rev.

[B14] Clarkson P, Celermajer DS, Donald AE, Sampson M, Sorensen KE, Adams M, Yue DK, Betteridge DJ, Deanfield JE (1996). Impaired vascular reactivity in insulin-dependent diabetes mellitus is related to disease duration and low density lipoprotein cholesterol levels. J Am Coll Cardiol.

[B15] Taylor PD, Wickenden AD, Mirrlees DJ, Poston L (1994). Endothelial function in the isolated perfused mesentery and aortae of rats with streptozotocin-induced diabetes: effect of treatment with the aldose reductase inhibitor, ponalrestat. Br J Pharmacol.

[B16] Junod A, Lambert AE, Stauffacher W, Renold AE (1969). Diabetogenic Action of Streptozotocin: Relationship of Dose to Metabolic Response. J Clin Invest.

[B17] Casteels R, Kitamura K, Kuriyama H, Suzuki H (1977). Excitation-contraction coupling in the smooth muscle cells of the rabbit main pulmonary artery. J Physiol.

[B18] Leach RM, Twort CH, Cameron IR, Ward JP (1992). A comparison of the pharmacological and mechanical properties in vitro of large and small pulmonary arteries of the rat. Clin Sci.

[B19] Bian K, Doursout MF, Murad F (2008). Vascular system: role of nitric oxide in cardiovascular diseases. J Clin Hypertens.

[B20] Russ RD, Tobin BW (1998). Pancreatic islet transplantation, but not intensive insulin therapy, corrects the pulmonary vascular complications of streptozotocin diabetes. Can J Physiol Pharmacol.

[B21] Wascher TC, Graier WF, Bahadori B, Toplak H (1994). Time course of endothelial dysfunction in diabetes mellitus. Circulation.

[B22] Suzuki H, Twarog BM (1982). Membrane properties of smooth muscle cells in pulmonary arteries of the rat. Am J Physiol.

[B23] MacLean MR, Sweeney G, Baird M, McCulloch KM, Houslay M, Morecroft I (1996). 5-Hydroxytryptamine receptors mediating vasoconstriction in pulmonary arteries from control and pulmonary hypertensive rats. Br J Pharmacol.

[B24] Cameron NE, Cotter MA (1992). Impaired contraction and relaxation in aorta from streptozotocin-diabetic rats: role of polyol pathway. Diabetologia.

[B25] Chang KS, Stevens WC (1992). Endothelium-dependent increase in vascular sensitivity to phenylephrine in long-term streptozotocin diabetic rat aorta. Br J Pharmacol.

[B26] MacLean MR (1999). Pulmonary hypertension, anorexigens and 5-HT: pharmacological synergism in action?. Trends Pharmacol Sci.

[B27] Dempsie Y, Maclean MR (2008). Pulmonary hypertension: therapeutic targets within the serotonin system. Br J Pharmacol.

[B28] Barradas MA, Gill DS, Fonseca VA, Mikhailidis DP, Dandona P (1988). Intraplatelet serotonin in patients with diabetes mellitus and peripheral vascular disease. Eur J Clin Invest.

[B29] Martín FJ, Míguez JM, Aldegunde M, Atienza G (1995). Platelet serotonin transport is altered in streptozotocin-induced diabetic rats. Life Sci.

[B30] Petrisiæ MS, Augood SJ, Bicknell RJ (1997). Monoamine transporter gene expression in the central nervous system in diabetes mellitus. J Neurochem.

[B31] MacLean MR, Deuchar GA, Hicks MN, Morecroft I, Shen S, Sheward J, Colston J, Loughlin L, Nilsen M, Dempsie Y, Harmar A (2004). Overexpression of the 5-hydroxytryptamine transporter gene: effect on pulmonary hemodynamics and hypoxia-induced pulmonary hypertension. Circulation.

[B32] Wakabayashi I, Hatake K, Kimura N, Kakishita E, Nagai K (1987). Modulation of vascular tonus by the endothelium in experimental diabetes. Life Sci.

[B33] Oyama Y, Kawasaki H, Hattori Y, Kanno M (1986). Attenuation of endothelium-dependent relaxation in aorta from diabetic rats. Eur J Pharmacol.

[B34] Kamata K, Miyata N, Kasuya Y (1989). Impairment of endothelium-dependent relaxation and changes in levels of cyclic GMP in aorta from streptozotocin-induced diabetic rats. Br J Pharmacol.

[B35] Otter DJ, Chess-Williams R (1994). The effects of aldose reductase inhibition with ponalrestat on changes in vascular function in streptozotocin diabetic rats. Br J Pharmacol.

[B36] Utkan T, Sarioglu Y, Yildirim S (1998). Impaired contraction and relaxation in the aorta of streptozotocin-diabetic rats. Pharmacology.

[B37] Bhardwaj R, Moore PK (1988). Increased vasodilator response to acetylcholine of renal blood vessels from diabetic rats. J Pharm Pharmacol.

[B38] Taylor PD, McCarthy AL, Thomas CR, Poston L (1992). Endothelium-dependent relaxation and noradrenaline sensitivity in mesenteric resistance arteries of streptozotocin-induced diabetic rats. Br J Pharmacol.

[B39] Tawfik HE, El-Remessy AB, Matragoon S, Ma G, Caldwell RB, Caldwell RW (2006). Simvastatin improves diabetes-induced coronary endothelial dysfunction. J Pharmacol Exp Ther.

